# No increase in new users of blood glucose-lowering drugs in Norway 2006–2011: a nationwide prescription database study

**DOI:** 10.1186/1471-2458-14-520

**Published:** 2014-05-29

**Authors:** Hanne Strøm, Randi Selmer, Kåre I Birkeland, Henrik Schirmer, Tore Julsrud Berg, Anne Karen Jenum, Kristian Midthjell, Christian Berg, Lars Christian Stene

**Affiliations:** 1 Department of Pharmacoepidemiology, Division of Epidemiology, Norwegian Institute of Public Health, P.O.Box 4404, Oslo N-0403, Norway; 2 Department of Endocrinology, Morbid Obesity and Preventive Medicine, Oslo University Hospital, Oslo, Norway; 3 Department of Clinical Medicine, University of Tromsø, Hansine Hansens veg 18, Tromsø 9019, Norway; 4 Division of Heart and Lung Disease, University Hospital of North Norway, Tromsø, Norway; 5 Faculty of Medicine, University of Oslo, Oslo, Norway; 6 Faculty of Health, Oslo and Akershus University College of Applied Science, Oslo, Norway; 7 Department of Public Health and General Practice, Norwegian University of Science and Technology, Trondheim, Norway

**Keywords:** Diabetes, Blood glucose-lowering drugs, Incidence, Prevalence, Prescription database, Norway

## Abstract

**Background:**

National estimates for the occurrence of diabetes are difficult to obtain, particularly time trends in incidence. The aim was to describe time trends in prevalent and incident use of blood glucose-lowering drugs by age group and gender in Norway during 2005–2011.

**Methods:**

Data were obtained from the nationwide Norwegian Prescription Database. We defined prevalent users of “insulins only” as individuals having no oral antidiabetic drugs (OAD) dispensed from a pharmacy during the previous 24 months or in the subsequent 12 months. Incident users had no blood glucose-lowering drugs dispensed in the previous 24 months; incident “insulins only” users also had no OAD in the subsequent 12 months.

**Results:**

In 2011, 3.2% of the population had blood glucose-lowering drugs dispensed, and the incidence rate was 313 per 100,000 person years. The prevalence of OAD use increased from 1.8% in 2005 to 2.4% in 2011; however a decreasing trend in incidence of OAD use was observed, particularly in those aged 70 years and older. In 2010, 0.64% of the population had insulins only dispensed, with an overall incidence rate in the total population of 33 per 100,000 person years which was stable over time.

**Conclusions:**

In this nationwide study, we found that although the prevalent use of OAD had increased in recent years, incident use was stable or had decreased. This may indicate that the increase in diabetes occurrence in Norway is levelling off, at least temporarily.

## Background

Diabetes constitutes a major public health challenge and numerous studies suggest that the prevalence is increasing in most countries [1]. However, reliable national estimates for the occurrence of diabetes are difficult to obtain, particularly time trends in incidence. Population-based health studies have limitations, since study samples tend to have low and therefore potentially biased participation [2,3]. A few countries such as Denmark, Sweden and Scotland have established national diabetes registers to monitor trends in the occurrence of diabetes [4,5], but few of these have complete coverage.

While most approaches to estimating the total number with diagnosed diabetes in a population are likely to have important sources of error, data from nationwide prescription drug databases can overcome the problems with biased participation and limited sample sizes to estimate gender- and age-specific trends. All diagnosed patients with Type 1 diabetes and a large majority of patients with Type 2 diabetes in most Western countries are treated with blood glucose-lowering drugs. In European children, insulin use essentially indicates a diagnosis of Type 1 diabetes [6].

Blood glucose-lowering drugs are used for other indications than diabetes, e.g. metformin for the treatment of polycystic ovary syndrome (PCOS) and this has to be taken into account. Furthermore, diabetes not treated with blood glucose-lowering drugs and undiagnosed diabetes will not be covered but important trends can nevertheless be monitored with high quality population-based prescription databases [7].

The primary aim of this study was to describe time trends in prevalent and incident use of blood glucose-lowering drugs by age group and gender in Norway during 2005–2011.

## Methods

### Data sources

The Norwegian Prescription Database (NorPD) was established in 2004 and includes all prescriptions redeemed by individual patients (encrypted) at pharmacies. Information on drug use in hospitals and nursing homes is collected but not on an individual level. NorPD covers drug supply for the entire population of Norway (4.92 million inhabitants in 2011). All pharmacies are obliged by law to report data electronically to the NorPD each month [8]. Blood glucose-lowering drugs are classified in the Anatomical Therapeutic Chemical (ATC) classification system in group A10. Insulins and analogues are classified in A10A, while other blood glucose-lowering drugs, referred to here as oral antidiabetic drugs (OAD), are classified in A10B [9,10].

Research was part of the mandate sanctioned by the Norwegian Data Protection Authority when the NorPD was established. Permission to conduct this descriptive study was therefore not required from the Regional Committee for Medical Research Ethics.

### Study population

The size of the population by gender and age in each calendar year studied was obtained from Statistics Norway. The analyses were based on data from NorPD about drugs prescribed and dispensed from 1 January 2004 to 31 December 2011. Data from 2004 were included to allow for a run-in period. Treatment with blood glucose-lowering drugs was used as a proxy for drug-treated diabetes.

### Outcome measures

**Figure 1:**
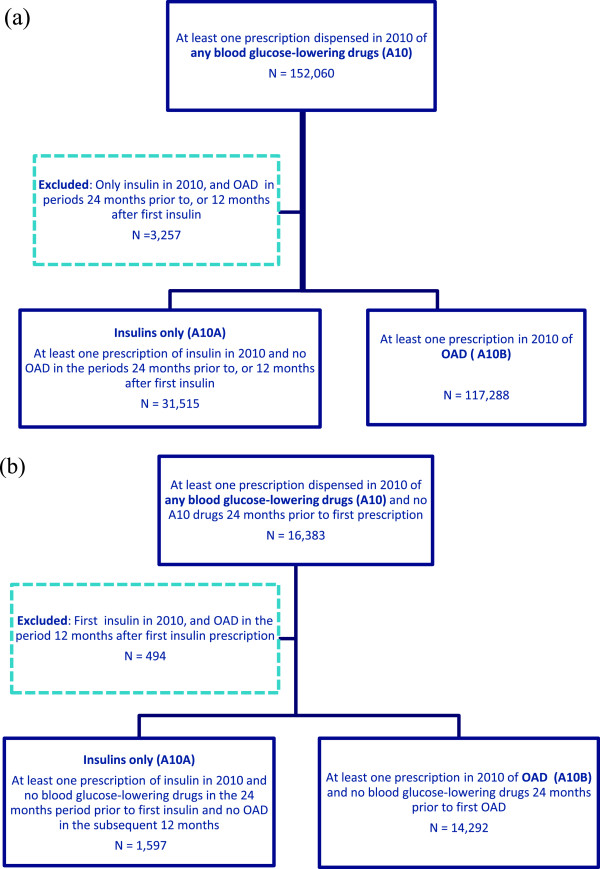
**Flow chart illustrating the definitions used for classifying subjects as (a) prevalent and (b) incident users of any blood glucose-lowering drug, OAD and insulins only.** Figures for 2010 are shown.

Figure 1 illustrates the definitions used for classifying subjects as (a) prevalent and (b) incident users of any blood glucose-lowering drug, OAD and insulins only.

(a) Prevalence outcome measures

Individuals with at least one prescription of any blood glucose-lowering drug dispensed in a calendar year were counted as prevalent users. Individuals with at least one OAD prescription alone or in combination with insulins dispensed in a calendar year were counted as prevalent users of OAD. Individuals with at least one prescription of insulins dispensed in a calendar year but no OAD in the periods 24 months prior to or 12 months after their first insulin prescription was dispensed were counted as prevalent users of insulins only.

(b) Incidence outcome measures

Incident users of blood glucose-lowering drugs were defined as individuals having at least one prescription of these drugs dispensed in a calendar year but not in the 24 months period prior to the first prescription. Incident users of OAD were defined as individuals having at least one prescription of OAD dispensed in a calendar year or period but no blood glucose-lowering drugs in the previous 24 months. Incident users of insulins only were defined as individuals having at least one prescription of insulin dispensed in a calendar year or period but no blood glucose-lowering drugs in the previous 24 months, and no OAD in the subsequent 12 months.

### Statistical methods

Person years for calculation of incidence rates were estimated from the mean population size for each gender, age group and calendar year. Influence of age and gender and test for trend by calendar year were modelled by Poisson regression using STATA version 13 (StataCorp LP, College Station, TX, USA). Incidence rate ratio (IRR) per year was calculated, and interaction between calendar year and age was tested by likelihood ratio test.

## Results

### Prevalent use of blood glucose-lowering drugs

**Figure 2:**
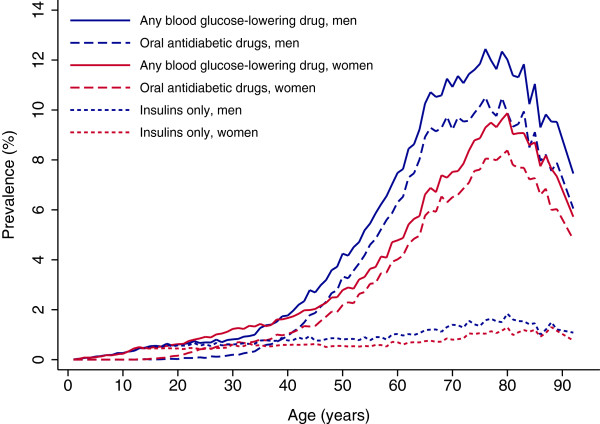
**Prevalent use of blood glucose-lowering drugs by age and gender in Norway in 2010.** Solid lines: any blood glucose-lowering drug (A10). Dashed lines, oral antidiabetic drugs. Short dashed lines: insulins only; i.e. individuals having insulins dispensed in 2010, but no OAD in the previous 24 months or in the subsequent 12 months after the first prescription filled.

**Figure 3:**
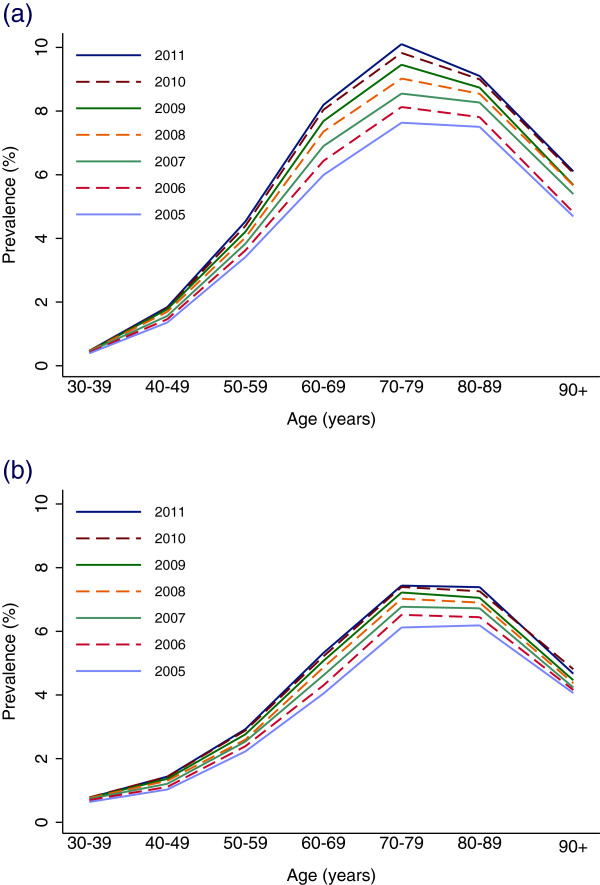
Time trends in prevalence of OAD (A10B) use by 10 year age groups in (a) men and (b) women.

In 2011, 156,540 individuals (3.2% of the population) had blood glucose-lowering drugs dispensed compared to 117,541 individuals (2.5% of the population) in 2005. As expected, the prevalence increased strongly with age. The prevalence was higher in men than in women after 40 years of age. The peak prevalence in men was at age 76 years (12.4%) and in women at age 80 years (9.9%), a pattern very similar to that of users of OAD (Figure 2).The prevalence of OAD use increased from 1.8% in 2005 to 2.4% in 2011 and there was an increase in all age groups, with a larger increase in men than in women (Figure 3).

In 2010, 31,515 individuals (0.64% of the population) had insulins only dispensed and the prevalence was stable during the period 2006–2010 (data not shown). In a sensitivity analysis we calculated the prevalent users of insulins only by extending the period without previous use of OAD up to six years. With a six year period, the number declined to 27,927 (0.57%) (Additional file 1: Table S1).

### Incident use of blood glucose-lowering drugs

**Figure 4:**
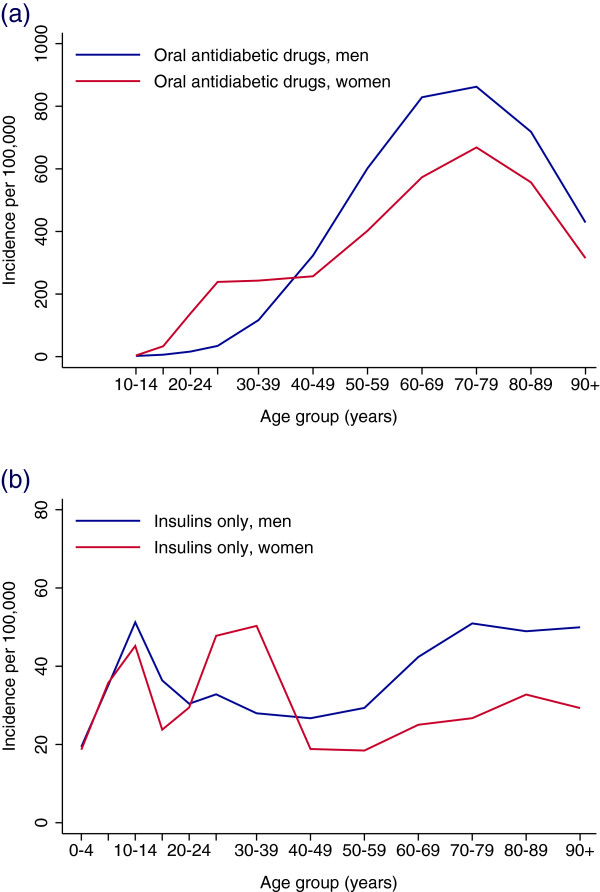
**Incident use of blood glucose-lowering drugs by age and gender in Norway.** Incident use is defined as no use of blood glucose-lowering drugs in the previous 24 months. **(a)** OAD (A10B); period 2006–2011. **(b)** Insulins and analogues (A10A); period 2006–2010. No OAD dispensed in the subsequent 12 months.

**Figure 5:**
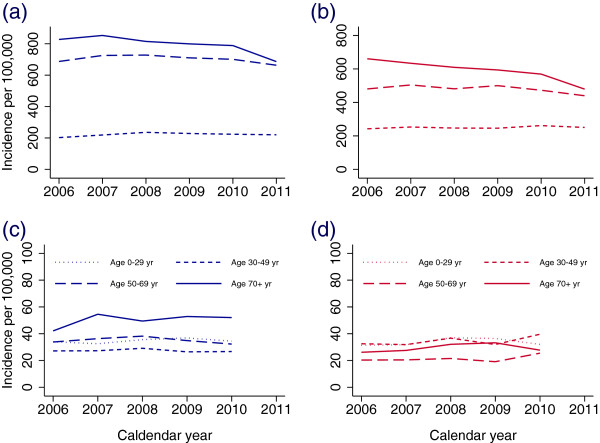
**Time trends in incident use of blood glucose-lowering drugs in Norway.** Incident defined as no use of blood glucose-lowering drugs in the previous 24 months. In addition for incident use of insulins only, no OAD dispensed in the subsequent 12 months. **(a)** Incident use of OAD (A10B) in men (**b)** Incident use of OAD in women. **(c)** Incident use of insulins only (A10A) in men (**d)** Incident use of insulins only in women.

The incidence of OAD use was significantly higher in women than in men aged 20–39 years and the pattern was reversed after 40 years of age (Figure 4a). In 2011, the overall incidence rate was significantly lower than the previous year (Table 1). The decreasing incidence of OAD use during the study period was largely confined to the 70 years or older age group (IRR per year = 0.97 (95% CI 0.96-0.98) in men and IRR = 0.95 (95% CI 0.94-0.96) in women) but a weaker significant decreasing trend was also observed for those aged 50–69 years (IRR per year = 0.99 (95% CI 0.98-1.00) in men and IRR = 0.98 (95% CI 0.97-0.99) in women) (Figures 5a-b).

**Table 1:** Incident users of any blood glucose-lowering drug, oral antidiabetic drugs (OAD) and insulins only*

		**Any blood glucose-lowering drug (A10)**			**OAD (A10B)**			**Insulins only (A10A only)**		
**Year**	**Person years**	**No of patients**	**Incidence rate per 100,000 person years**	**95% confidence interval**	**No of patients**	**Incidence rate per 100,000 person years**	**95% confidence interval**	**No of patients**	**Incidence rate per 100,000 person years**	**95% confidence interval**
**Women**										
2006	2,347,036	7,531	320	(314, 328)	6,752	288	(281, 295)	670	29	(26, 31)
2007	2,366,461	7,769	328	(321, 336)	6,927	293	(286, 300)	679	29	(27, 31)
2008	2,390,716	7,677	321	(314, 328)	6,717	281	(274, 288)	783	33	(31, 35)
2009	2,418,595	7,832	323	(317, 331)	6,912	286	(279, 293)	745	31	(29, 33)
2010	2,445,249	7,778	318	(311, 325)	6,817	279	(272, 286)	784	32	(30, 34)
2011	2,473,228	7,269	293	(287, 301)	6,369	258	(251, 264)	†		
**Men**										
2006	2,314,006	7,829	338	(331, 346)	6,830	295	(288, 302)	756	33	(30, 35)
2007	2,342,823	8,374	357	(350, 365)	7,326	313	(306, 320)	793	34	(32, 36)
2008	2,377,361	8,655	364	(356, 372)	7,498	315	(308, 323)	845	36	(33, 38)
2009	2,411,205	8,620	357	(350, 365)	7,453	309	(302, 316)	839	35	(33, 37)
2010	2,443,697	8,605	352	(345, 360)	7,475	306	(299, 313)	813	33	(31, 36)
2011	2,479,989	8,246	332	(325, 340)	7,131	288	(281, 294)	†		
**Total**										
2006	4,661,041	15,360	329	(324, 334)	13,582	291	(287, 297)	1,426	31	(29, 32)
2007	4,709,284	16,143	342	(338, 348)	14,253	303	(298, 308)	1,472	31	(30, 33)
2008	4,768,077	16,332	342	(337, 348)	14,215	298	(293, 303)	1,628	34	(33, 36)
2009	4,829,800	16,452	340	(335, 346)	14,365	297	(293, 302)	1,584	33	(31, 34)
2010	4,888,946	16,383	335	(330, 340)	14,292	292	(288, 297)	1,597	33	(31, 34)
2011	4,953,217	15,515	313	(308, 318)	13,500	273	(268, 277)	†		

^*^Incident use is defined as no use of blood glucose-lowering drugs in the previous 24 months. In addition, for insulins only no OAD was dispensed in the subsequent 12 months.

^†^For insulins only users there are no data for 2011 due to the definition applied, requiring a 12 month follow up period after first insulin prescription filled.

In women, the highest incidence of insulin use only was in the 30–39 year age group, and for men it was highest among the 70–79 year age group (Figure 4b). In a sensitivity analysis, 59% of incident insulins only users among women aged 30–39 years in 2008 had stopped insulin treatment within the following two years, indicating gestational diabetes rather than Type 1 diabetes (Additional file 2: Figure S1). There was no significant overall time trend in incident use of insulins only for men (IRR per year = 1.01 (95% CI (0.98-1.03) but a weak increasing overall trend for women was observed (IRR per year = 1.03 (1.01-1.05) (Figures 5c-d).

## Supplementary material

Detailed data tabulated by age group, gender and calendar year are provided in Additional file 1: Table S2-S5. Population sizes used to calculate incidence and prevalence are shown in Additional file 1: Table S6.

## Discussion

We have monitored the use of blood glucose-lowering drugs in Norway, and found an increase in the overall prevalence from 2.5% in 2005 to 3.2% in 2011. Interestingly, the number of new users was, for the first time in the period, significantly lower in 2011 than the year before and this was primarily due to a reduction in incident use of OAD in the 70 years and older age groups.

The prevalence of diabetes depends on the incidence and mean duration of the disease, both of which are affected by several factors. For example, increased survival among those with the diagnosis will increase the prevalence [11] and higher life expectancy (ageing) in the general population could influence the incidence. Others have shown that declining mortality among patients may contribute, but that increasing incidence was the main driver of the increasing prevalence over time, at least in Denmark 1992–2003 [7] and in Scotland 1993–2004 [5]. We did not have mortality data in our study but others have shown a secular decrease in mortality from ischaemic heart disease among patients with diabetes in Norway [12], suggesting that this could have contributed to the observed increasing prevalence with concurrent decline in incidence.

The strength of this study is the complete coverage of the Norwegian population [8]. NorPD should therefore provide good estimates for the number of patients with diabetes treated with blood glucose-lowering drugs (100% reimbursed), with a few caveats that are discussed below. The study does not cover diabetes that is not treated with blood glucose-lowering medication, and classification of diabetes into Type 1 or Type 2 diabetes based on available data is difficult in the older age groups. Individuals with at least one prescription of blood glucose-lowering drugs dispensed were included but the vast majority had actually had several prescriptions. In 2010, of incident users in both groups (insulins only and OAD) recorded, 88% redeemed more than one prescription during the following 12 months. Institutionalisation, emigration, switch to lifestyle treatment only, or off-label use (PCOS, obesity) could partly confound the results (see below). The high incident use of insulins in women in reproductive age might be explained by gestational diabetes.

Another limitation is lack of individual level data of institutionalised patients with diabetes. About 13% of the population above 80 years of age (approximately 30,000 people) lived in nursing homes in Norway in 2011 (figures from *Statistics Norway*, http://www.ssb.no). Thus, the prevalence and incidence in the oldest patient groups will be underestimated. For instance, recalculating for users of blood glucose-lowering drugs in 2011, the prevalence increased from 9.0% to 10.4% in the over 80 age group. However, data from the NorPD show that in 2011 only 2.4% of total DDDs (Defined Daily Doses) [10] prescribed of blood glucose-lowering drugs were dispensed to institutions. For insulins, 3.4% of total DDDs prescribed were dispensed to institutions. This is probably too little to have any major influence on our observed time trends.

Migration of patients has not been taken into account in the study. In principle, a prevalent user of blood glucose-lowering drugs, moving from another country to Norway, may therefore have been mistakenly counted as an incident user. Again, this is likely to be a minor source of error, particularly for time trends.

The use of dispensed drugs in a prescription database as a proxy for diabetes will obviously result in an underestimation of the total number of patients with diabetes in the population, as a considerable proportion of Type 2 diabetes patients are treated with lifestyle measures alone. Studies from Norway in the period 1995–2005 report that 20-35% of prevalent patients are treated by diet only [13-15]. Data from 33 general practices in two representative areas of Norway showed a decline in the proportion of patients with diabetes not treated with blood glucose-lowering drugs, from 30.7% in 1995 to 28.4% in 2005 [14]. Data from the Swedish Diabetes Register show a decline in the proportion of patients with Type 2 diabetes treated with diet only from 25.3% in 2008 to 23.4% in 2011 [16]. We do not have nationally representative data on the proportion of diabetes patients not treated with blood glucose-lowering medication for our study period (2005–2011) but if there is a real decline in this proportion, it is particularly interesting that the incident use of blood glucose-lowering drugs did not increase during the study period. The proportion of patients not treated may also be influenced by the number of new blood glucose-lowering drugs available on the market promoted by the pharmaceutical industry.

On the other hand, we cannot rule out the possibility that the proportion of diagnosed Type 2 diabetes patients treated with OAD has declined during the study period; for instance, as a result of patients being diagnosed earlier and increasing focus on beneficial dietary changes. Safety concerns in the elderly regarding polypharmacy and hypoglycaemic episodes could also contribute to such a trend.

The large majority of patients receiving OAD are likely to have Type 2 diabetes. On the other hand, there will be some misclassification from prescriptions for patients with PCOS, pre-diabetes and the metabolic syndrome [17-19]. Some women of reproductive age who are treated with metformin are probably suffering from PCOS rather than diabetes. In 2011, the total number of women in this age group treated with metformin was 3,482 (0.54% in this age group population), and a total of 3,712 (0.58%) received oral blood glucose-lowering drugs. In European studies, the occurrence of PCOS has been reported to be 6.5-8% [20].

Since insulins and analogues are used by both patients with Type 1 diabetes and Type 2 diabetes, the definition applied in the outcome measure “prevalent user of insulins only” is aimed at estimating a group of patients with Type 1 diabetes. Some of the defined insulins only users may have received treatment with OAD prior to the 24 month period, as suggested in our sensitivity analysis that extends the period to six years. This could be explained by switching from OAD to insulins only e.g. due to the relative contraindication of OAD in severe heart and kidney failure. Thus, the estimated prevalence of Type 1 diabetes in the population in this study may be overestimated to some extent in the elderly.

There was a stable incidence of use of insulins only. Recent data on childhood-onset Type 1 diabetes from the Norwegian Childhood Diabetes Registry show little or no increase in the past few years, which is consistent with the current results [6]. In the only previous Norwegian study of Type 1 diabetes incidence in individuals over 15 years of age from Norway, Joner & Søvik estimated an incidence of 17/100,000 person years in the age group 15–29 years during 1978–1982 [21]. The current study showed 32/100,000 person years across all age groups. In the age groups over 30–40 years, diabetes classification becomes more challenging. It is nevertheless clear that a substantial proportion of incident Type 1 diabetes cases arise in adulthood [22].

## Conclusions

There was no increase in the incident use of blood glucose-lowering drugs in Norway in the period 2006–2011 and there was even a decline in the incident use of OAD, particularly in those aged 70 years and older. This occurred despite an increase in prevalent use of OAD. The prevalence and incidence of insulin use only was stable during the study period. This may indicate that the increase in diabetes occurrence in Norway is levelling off, at least temporarily.

## Abbreviations

ATC: Anatomical therapeutic chemical; DDD: Defined daily dose; NorPD: Norwegian prescription database; OAD: Oral antidiabetic drugs; PCOS: Polycystic ovary syndrome.

## Competing interests

The authors declare that they have no competing interests.

## Authors’ contributions

HS conceived the study and drafted and edited the manuscript. KIB, HSc, TJB, AKJ, KM, CB and LCS developed the design, interpreted the results and revised the manuscript. RS participated in the design of the study and performed the statistical analysis. All authors reviewed the manuscript for intellectual content and approved the final manuscript.

## Pre-publication history

The pre-publication history for this paper can be accessed here:

http://www.biomedcentral.com/1471-2458/14/520/prepub

## Supplementary Material

Additional file 1: Table S1 Effect of extending the period for: * no previous use of oral antidiabetic drugs (A10B) on estimated number of prevalent users in 2010; ** no previous use of blood glucose-lowering drugs (A10) on estimated number of incident users in 2010. **Table S2.** Prevalent users of blood glucose-lowering drugs (A10) in Norway 2005-2011. **Table S3.** Prevalent users of oral antidiabetic drugs (A10B) in Norway 2005-2011. **Table S4.** Incident users and incidence rate (per 100,000 person years) of use of oral antidiabetic drugs (A10B) in Norway 2006-2011 (no A10 previous 24 months). **Table S5.** Incident users and incidence rate (per 100,000 person years) of insulin only use 2006-2010 in Norway (no A10 previous 24 months and no A10B 12 months after first insulin prescription dispensed). **Table S6.** Mean population used in calculation of incidence rates and prevalences of use of blood glucose-lowering drugs in Norway 2005-2011. Click here for file

Additional file 2: Figure S1 New users of insulins only among women aged 30-39 years in 2008. Click here for file
