# Supplementary material for: No increase in new users of blood glucose-lowering drugs in Norway 2006–2011: a nationwide prescription database study
Source: BMC Public Health. 2014 May 29;14:520. doi: 10.1186/1471-2458-14-520 (PMC4045953; doi:10.1186/1471-2458-14-520)
Supplement: Additional file 1: Table S1 — Effect of extending the period for: * no previous use of oral antidiabetic drugs (A10B) on estimated number of prevalent users in 2010; ** no previous use of blood glucose-lowering drugs (A10) on estimated number of incident users in 2010. Table S2. Prevalent users of blood glucose-lowering drugs (A10) in Norway 2005-2011. Table S3. Prevalent users of oral antidiabetic drugs (A10B) in Norway 2005-2011. Table S4. Incident users and incidence rate (per 100,000 person years) of use of oral antidiabetic drugs (A10B) in Norway 2006-2011 (no A10 previous 24 months). Table S5. Incident users and incidence rate (per 100,000 person years) of insulin only use 2006-2010 in Norway (no A10 previous 24 months and no A10B 12 months after first insulin prescription dispensed). Table S6. Mean population used in calculation of incidence rates and prevalences of use of blood glucose-lowering drugs in Norway 2005-2011. [file 1471-2458-14-520-S1.docx]

**Additional file 1: Table S1**

| Effect of extending the period for  * no previous use of oral antidiabetic drugs (A10B) on estimated number of prevalent users in 2010  **no previous use of blood glucose-lowering drugs (A10) on estimated number of incident users in 2010 | | |
| --- | --- | --- |
| Reference period (years) | Prevalent users of insulin only* | Incident users of insulin only** |
| 1 | 32,921 | 2,311 |
| 2 | 31,515 | 1,597 |
| 3 | 30,343 | 1,508 |
| 4 | 29,429 | 1,463 |
| 5 | 28,635 | 1,437 |
| 6 | 27,927 | 1,427 |

| **Table S2**  Prevalent users of blood glucose-lowering drugs (A10) in Norway 2005-2011 | | | | | | | | | | | |  |  |  |
| --- | --- | --- | --- | --- | --- | --- | --- | --- | --- | --- | --- | --- | --- | --- |
| Age | 2005 | | 2006 | | 2007 | | 2008 | | 2009 | | 2010 | | 2011 | |
| (years) | N | % | N | % | N | % | N | % | N | % | N | % | N | % |
| **Men** |  |  |  |  |  |  |  |  |  |  |  |  |  |  |
| 0-4 | 66 | 0.1 | 58 | 0.0 | 51 | 0.0 | 64 | 0.0 | 65 | 0.0 | 63 | 0.0 | 70 | 0.0 |
| 5-9 | 252 | 0.2 | 266 | 0.2 | 262 | 0.2 | 260 | 0.2 | 271 | 0.2 | 273 | 0.2 | 283 | 0.2 |
| 10-14 | 546 | 0.3 | 565 | 0.4 | 582 | 0.4 | 585 | 0.4 | 608 | 0.4 | 608 | 0.4 | 593 | 0.4 |
| 15-19 | 778 | 0.5 | 808 | 0.5 | 832 | 0.5 | 860 | 0.5 | 896 | 0.5 | 940 | 0.6 | 968 | 0.6 |
| 20-29 | 1,777 | 0.6 | 1,846 | 0.6 | 1,936 | 0.7 | 2,027 | 0.7 | 2,106 | 0.7 | 2,205 | 0.7 | 2,285 | 0.7 |
| 30-39 | 4,088 | 1.2 | 4,095 | 1.2 | 4,146 | 1.2 | 4,161 | 1.2 | 4,107 | 1.2 | 4,118 | 1.2 | 4,054 | 1.2 |
| 40-49 | 7,290 | 2.2 | 7,803 | 2.3 | 8,358 | 2.4 | 9,034 | 2.6 | 9,537 | 2.7 | 9,846 | 2.7 | 10,168 | 2.7 |
| 50-59 | 13,518 | 4.4 | 14,029 | 4.6 | 14,726 | 4.8 | 15,405 | 5.0 | 16,156 | 5.2 | 16,880 | 5.4 | 17,556 | 5.5 |
| 60-69 | 15,246 | 7.4 | 17,215 | 7.8 | 19,138 | 8.2 | 21,094 | 8.7 | 22,659 | 9.0 | 24,291 | 9.3 | 25,537 | 9.5 |
| 70-79 | 12,390 | 9.4 | 12,969 | 9.9 | 13,574 | 10.3 | 14,291 | 10.8 | 15,139 | 11.2 | 15,924 | 11.6 | 16,538 | 11.9 |
| 80+ | 6,903 | 8.8 | 7,275 | 9.1 | 7,718 | 9.5 | 8,021 | 9.8 | 8,297 | 10.0 | 8,707 | 10.4 | 8,922 | 10.5 |
| Total | 62,854 | 2.7 | 66,929 | 2.9 | 71,323 | 3.0 | 75,802 | 3.2 | 79,841 | 3.3 | 83,855 | 3.4 | 86,974 | 3.5 |
| **Women** | |  |  |  |  |  |  |  |  |  |  |  |  |  |
| 0-4 | 38 | 0.0 | 38 | 0.0 | 49 | 0.0 | 52 | 0.0 | 56 | 0.0 | 59 | 0.0 | 59 | 0.0 |
| 5-9 | 268 | 0.2 | 268 | 0.2 | 254 | 0.2 | 227 | 0.2 | 219 | 0.2 | 233 | 0.2 | 239 | 0.2 |
| 10-14 | 519 | 0.3 | 538 | 0.4 | 553 | 0.4 | 587 | 0.4 | 614 | 0.4 | 604 | 0.4 | 590 | 0.4 |
| 15-19 | 649 | 0.4 | 716 | 0.5 | 750 | 0.5 | 757 | 0.5 | 806 | 0.5 | 827 | 0.5 | 845 | 0.5 |
| 20-29 | 2,292 | 0.8 | 2,392 | 0.9 | 2,442 | 0.9 | 2,518 | 0.9 | 2,579 | 0.9 | 2,641 | 0.9 | 2,654 | 0.8 |
| 30-39 | 4,155 | 1.2 | 4,338 | 1.3 | 4,459 | 1.3 | 4,509 | 1.4 | 4,489 | 1.4 | 4,558 | 1.4 | 4,496 | 1.4 |
| 40-49 | 5,078 | 1.6 | 5,451 | 1.7 | 5,916 | 1.8 | 6,386 | 1.9 | 6,737 | 2.0 | 7,092 | 2.1 | 7,268 | 2.1 |
| 50-59 | 8,475 | 2.9 | 8,935 | 3.0 | 9,358 | 3.1 | 9,642 | 3.2 | 10,214 | 3.4 | 10,692 | 3.5 | 10,961 | 3.6 |
| 60-69 | 10,458 | 4.9 | 11,565 | 5.2 | 12,797 | 5.4 | 13,910 | 5.7 | 14,858 | 5.9 | 15,698 | 6.0 | 16,466 | 6.1 |
| 70-79 | 12,046 | 7.4 | 12,424 | 7.8 | 12,744 | 8.0 | 13,133 | 8.3 | 13,511 | 8.5 | 13,819 | 8.6 | 13,939 | 8.6 |
| 80+ | 10,709 | 7.2 | 11,061 | 7.4 | 11,341 | 7.6 | 11,576 | 7.7 | 11,753 | 7.8 | 11,982 | 8.0 | 12,049 | 8.1 |
| Total | 54,687 | 2.3 | 57,726 | 2.5 | 60,663 | 2.6 | 63,297 | 2.6 | 65,836 | 2.7 | 68,205 | 2.8 | 69,566 | 2.8 |
| **Men+women** | |  |  |  |  |  |  |  |  |  |  |  |  |  |
| 0-4 | 104 | 0.0 | 96 | 0.0 | 100 | 0.0 | 116 | 0.0 | 121 | 0.0 | 122 | 0.0 | 129 | 0.0 |
| 5-9 | 520 | 0.2 | 534 | 0.2 | 516 | 0.2 | 487 | 0.2 | 490 | 0.2 | 506 | 0.2 | 522 | 0.2 |
| 10-14 | 1,065 | 0.3 | 1,103 | 0.4 | 1,135 | 0.4 | 1,172 | 0.4 | 1,222 | 0.4 | 1,212 | 0.4 | 1,183 | 0.4 |
| 15-19 | 1,427 | 0.5 | 1,524 | 0.5 | 1,582 | 0.5 | 1,617 | 0.5 | 1,702 | 0.5 | 1,767 | 0.5 | 1,813 | 0.6 |
| 20-29 | 4,069 | 0.7 | 4,238 | 0.8 | 4,378 | 0.8 | 4,545 | 0.8 | 4,685 | 0.8 | 4,846 | 0.8 | 4,939 | 0.8 |
| 30-39 | 8,243 | 1.2 | 8,433 | 1.2 | 8,605 | 1.3 | 8,670 | 1.3 | 8,596 | 1.3 | 8,676 | 1.3 | 8,550 | 1.3 |
| 40-49 | 12,368 | 1.9 | 13,254 | 2.0 | 14,274 | 2.1 | 15,420 | 2.2 | 16,274 | 2.3 | 16,938 | 2.4 | 17,436 | 2.4 |
| 50-59 | 21,993 | 3.6 | 22,964 | 3.8 | 24,084 | 4.0 | 25,047 | 4.1 | 26,370 | 4.3 | 27,572 | 4.4 | 28,517 | 4.5 |
| 60-69 | 25,704 | 6.1 | 28,780 | 6.5 | 31,935 | 6.8 | 35,004 | 7.2 | 37,517 | 7.4 | 39,989 | 7.7 | 42,003 | 7.8 |
| 70-79 | 24,436 | 8.3 | 25,393 | 8.7 | 26,318 | 9.1 | 27,424 | 9.4 | 28,650 | 9.7 | 29,743 | 10.0 | 30,477 | 10.1 |
| 80+ | 17,612 | 7.7 | 18,336 | 7.9 | 19,059 | 8.2 | 19,597 | 8.5 | 20,050 | 8.6 | 20,689 | 8.9 | 20,971 | 9.0 |
| Total | 117,541 | 2.5 | 124,655 | 2.7 | 131,986 | 2.8 | 139,099 | 2.9 | 145,677 | 3.0 | 152,060 | 3.1 | 156,540 | 3.2 |

| **Table S3**  Prevalent users of oral antidiabetic drugs (A10B) in Norway 2005-2011 | | | | | | | | | | | | | | |
| --- | --- | --- | --- | --- | --- | --- | --- | --- | --- | --- | --- | --- | --- | --- |
| Age | 2005 | | 2006 | | 2007 | | 2008 | | 2009 | | 2010 | | 2011 | |
|  | N | % | N | % | N | % | N | % | N | % | N | % | N | % |
| **Men** |  |  |  |  |  |  |  |  |  |  |  |  |  |  |
| 0-4 | 2 | 0.0 | 1 | 0.0 | 1 | 0.0 | 0 | 0.0 | 0 | 0.0 | 0 | 0.0 | 0 | 0.0 |
| 5-9 | 4 | 0.0 | 1 | 0.0 | 2 | 0.0 | 2 | 0.0 | 3 | 0.0 | 1 | 0.0 | 3 | 0.0 |
| 10-14 | 5 | 0.0 | 9 | 0.0 | 11 | 0.0 | 3 | 0.0 | 9 | 0.0 | 11 | 0.0 | 5 | 0.0 |
| 15-19 | 18 | 0.0 | 12 | 0.0 | 27 | 0.0 | 31 | 0.0 | 32 | 0.0 | 32 | 0.0 | 45 | 0.0 |
| 20-29 | 142 | 0.1 | 167 | 0.1 | 192 | 0.1 | 226 | 0.1 | 253 | 0.1 | 292 | 0.1 | 303 | 0.1 |
| 30-39 | 1,370 | 0.4 | 1,454 | 0.4 | 1,531 | 0.4 | 1,575 | 0.5 | 1,547 | 0.5 | 1,590 | 0.5 | 1,617 | 0.5 |
| 40-49 | 4,526 | 1.4 | 4,942 | 1.5 | 5,395 | 1.6 | 5,977 | 1.7 | 6,393 | 1.8 | 6,615 | 1.8 | 6,853 | 1.8 |
| 50-59 | 10,489 | 3.4 | 11,099 | 3.6 | 11,769 | 3.8 | 12,449 | 4.0 | 13,147 | 4.2 | 13,862 | 4.4 | 14,499 | 4.5 |
| 60-69 | 12,384 | 6.0 | 14,202 | 6.4 | 16,061 | 6.9 | 17,908 | 7.4 | 19,429 | 7.7 | 21,004 | 8.0 | 22,114 | 8.2 |
| 70-79 | 10,063 | 7.6 | 10,657 | 8.1 | 11,251 | 8.5 | 11,970 | 9.0 | 12,740 | 9.5 | 13,490 | 9.8 | 14,074 | 10.1 |
| 80+ | 5,660 | 7.2 | 5,996 | 7.5 | 6,431 | 7.9 | 6,699 | 8.2 | 6,917 | 8.3 | 7,204 | 8.6 | 7,363 | 8.7 |
| Total | 44,663 | 1.9 | 48,540 | 2.1 | 52,671 | 2.2 | 56,840 | 2.4 | 60,470 | 2.5 | 64,101 | 2.6 | 66,876 | 2.7 |
| **Women** |  |  |  |  |  |  |  |  |  |  |  |  |  |  |
| 0-4 | 2 | 0.0 | 1 | 0.0 | 1 | 0.0 | 1 | 0 | 0 | 0.0 | 0 | 0.0 | 0 | 0.0 |
| 5-9 | 2 | 0.0 | 1 | 0.0 | 2 | 0.0 | 1 | 3 | 3 | 0.0 | 3 | 0.0 | 2 | 0.0 |
| 10-14 | 18 | 0.0 | 19 | 0.0 | 11 | 0.0 | 11 | 9 | 9 | 0.0 | 9 | 0.0 | 14 | 0.0 |
| 15-19 | 101 | 0.1 | 109 | 0.1 | 124 | 0.1 | 102 | 106 | 106 | 0.1 | 118 | 0.1 | 103 | 0.1 |
| 20-29 | 972 | 0.4 | 1,067 | 0.4 | 1,124 | 0.4 | 1,117 | 1,151 | 1151 | 0.4 | 1,194 | 0.4 | 1,188 | 0.4 |
| 30-39 | 2,181 | 0.6 | 2,367 | 0.7 | 2,463 | 0.7 | 2,531 | 2,525 | 2525 | 0.8 | 2,575 | 0.8 | 2,529 | 0.8 |
| 40-49 | 3,309 | 1.0 | 3,605 | 1.1 | 3,974 | 1.2 | 4,359 | 4,644 | 4644 | 1.4 | 4,949 | 1.4 | 5,049 | 1.4 |
| 50-59 | 6,626 | 2.2 | 7,103 | 2.4 | 7,546 | 2.5 | 7,784 | 8,352 | 8352 | 2.8 | 8,795 | 2.9 | 9,030 | 2.9 |
| 60-69 | 8,566 | 4.0 | 9,659 | 4.3 | 10,877 | 4.6 | 11,908 | 12,826 | 12826 | 5.1 | 13,582 | 5.2 | 14,258 | 5.3 |
| 70-79 | 9,899 | 6.1 | 10,394 | 6.5 | 10,741 | 6.8 | 11,127 | 11,520 | 11520 | 7.2 | 11,885 | 7.4 | 12,025 | 7.4 |
| 80+ | 8,683 | 5.8 | 9,075 | 6.0 | 9,396 | 6.3 | 9,630 | 9,830 | 9830 | 6.5 | 10,077 | 6.7 | 10,132 | 6.8 |
| Total | 40,359 | 1.7 | 43,400 | 1.8 | 46,259 | 2.0 | 48,571 | 50,966 | 50966 | 2.1 | 53,187 | 2.2 | 54,330 | 2.2 |
| **Men+women** | |  |  |  |  |  |  |  |  |  |  |  |  |  |
| 0-4 | 4 | 0.0 | 2 | 0.0 | 2 | 0.0 | 1 | 0.0 | 0 | 0.0 | 0 | 0.0 | 0 | 0.0 |
| 5-9 | 6 | 0.0 | 2 | 0.0 | 4 | 0.0 | 3 | 0.0 | 6 | 0.0 | 4 | 0.0 | 5 | 0.0 |
| 10-14 | 23 | 0.0 | 28 | 0.0 | 22 | 0.0 | 14 | 0.0 | 18 | 0.0 | 20 | 0.0 | 19 | 0.0 |
| 15-19 | 119 | 0.0 | 121 | 0.0 | 151 | 0.0 | 133 | 0.0 | 138 | 0.0 | 150 | 0.0 | 148 | 0.0 |
| 20-29 | 1,114 | 0.2 | 1,234 | 0.2 | 1,316 | 0.2 | 1,343 | 0.2 | 1,404 | 0.2 | 1,486 | 0.2 | 1,491 | 0.2 |
| 30-39 | 3,551 | 0.5 | 3,821 | 0.6 | 3,994 | 0.6 | 4,106 | 0.6 | 4,072 | 0.6 | 4,165 | 0.6 | 4,146 | 0.6 |
| 40-49 | 7,835 | 1.2 | 8,547 | 1.3 | 9,369 | 1.4 | 10,336 | 1.5 | 11,037 | 1.6 | 11,564 | 1.6 | 11,902 | 1.6 |
| 50-59 | 17,115 | 2.8 | 18,202 | 3.0 | 19,315 | 3.2 | 20,233 | 3.3 | 21,499 | 3.5 | 22,657 | 3.7 | 23,529 | 3.7 |
| 60-69 | 20,950 | 5.0 | 23,861 | 5.4 | 26,938 | 5.8 | 29,816 | 6.1 | 32,255 | 6.4 | 34,586 | 6.6 | 36,372 | 6.8 |
| 70-79 | 19,962 | 6.8 | 21,051 | 7.2 | 21,992 | 7.6 | 23,097 | 7.9 | 24,260 | 8.2 | 25,375 | 8.5 | 26,099 | 8.7 |
| 80+ | 14,343 | 6.3 | 15,071 | 6.5 | 15,827 | 6.8 | 16,329 | 7.0 | 16,747 | 7.2 | 17,281 | 7.4 | 17,495 | 7.5 |
| Total | 85,022 | 1.8 | 91,940 | 2.0 | 98,930 | 2.1 | 105,411 | 2.2 | 111,436 | 2.3 | 117,288 | 2.4 | 121,206 | 2.4 |

| **Table S4**  Incident users and incidence rate (per 100,000 person years) of use of oral antidiabetic drugs (A10B) in Norway 2006-2011 (no A10 previous 24 months) | | | | | | | | | | | | | | |
| --- | --- | --- | --- | --- | --- | --- | --- | --- | --- | --- | --- | --- | --- | --- |
|  | 2006 | | 2007 | | 2008 | | 2009 | | 2010 | | 2011 | | Total | |
| Age (years) | N | Incidence rate | N | Incidence rate | N | Incidence rate | N | Incidence rate | N | Incidence rate | N | Incidence rate | N | Incidence rate |
| **Men** |  |  |  |  |  |  |  |  |  |  |  |  |  |  |
| 0-4 | 0 | 0 | 0 | 0 | 0 | 0 | 0 | 0 | 0 | 0 | 0 | 0 | 0 | 0 |
| 5-9 | 0 | 0 | 1 | 1 | 0 | 0 | 2 | 1 | 0 | 0 | 2 | 1 | 5 | 1 |
| 10-14 | 5 | 3 | 6 | 4 | 0 | 0 | 4 | 2 | 4 | 2 | 3 | 2 | 22 | 2 |
| 15-19 | 5 | 3 | 12 | 7 | 9 | 6 | 10 | 6 | 9 | 5 | 17 | 10 | 62 | 6 |
| 20-29 | 65 | 23 | 68 | 23 | 76 | 25 | 77 | 25 | 94 | 30 | 84 | 26 | 464 | 25 |
| 30-39 | 389 | 112 | 411 | 119 | 420 | 122 | 387 | 113 | 399 | 116 | 409 | 119 | 2,415 | 117 |
| 40-49 | 996 | 295 | 1,099 | 319 | 1,224 | 347 | 1,220 | 339 | 1,185 | 325 | 1,167 | 314 | 6,891 | 323 |
| 50-59 | 1,739 | 568 | 1,917 | 624 | 1,909 | 618 | 1,901 | 609 | 1,885 | 597 | 1,904 | 595 | 11,255 | 602 |
| 60-69 | 1,882 | 853 | 1,998 | 859 | 2,113 | 868 | 2,113 | 836 | 2,157 | 826 | 2,007 | 744 | 12,270 | 829 |
| 70-79 | 1,143 | 872 | 1,225 | 931 | 1,160 | 874 | 1,208 | 897 | 1,160 | 845 | 1,060 | 761 | 6,956 | 862 |
| 80+ | 606 | 755 | 589 | 726 | 587 | 718 | 531 | 641 | 582 | 696 | 478 | 565 | 3,373 | 682 |
| Total | 6,830 | 295 | 7,326 | 313 | 7,498 | 315 | 7,453 | 309 | 7,475 | 306 | 7,131 | 288 | 43,713 | 304 |
| **Women** |  |  |  |  |  |  |  |  |  |  |  |  |  |  |
| 0-4 | 0 | 0 | 0 | 0 | 0 | 0 | 0 | 0 | 0 | 0 | 0 | 0 | 0 | 0 |
| 5-9 | 0 | 0 | 1 | 1 | 0 | 0 | 1 | 1 | 0 | 0 | 1 | 1 | 3 | 0 |
| 10-14 | 10 | 7 | 4 | 3 | 4 | 3 | 5 | 3 | 5 | 3 | 7 | 5 | 35 | 4 |
| 15-19 | 54 | 36 | 57 | 37 | 39 | 25 | 47 | 30 | 59 | 38 | 52 | 33 | 308 | 33 |
| 20-29 | 532 | 191 | 545 | 193 | 534 | 184 | 595 | 200 | 552 | 180 | 581 | 184 | 3,339 | 189 |
| 30-39 | 825 | 244 | 804 | 241 | 784 | 236 | 777 | 236 | 826 | 251 | 820 | 250 | 4,836 | 243 |
| 40-49 | 778 | 240 | 872 | 266 | 857 | 257 | 870 | 256 | 937 | 271 | 881 | 251 | 5,195 | 257 |
| 50-59 | 1,213 | 408 | 1,216 | 409 | 1,162 | 388 | 1,292 | 428 | 1,234 | 405 | 1,160 | 376 | 7,277 | 402 |
| 60-69 | 1,292 | 576 | 1,471 | 625 | 1,456 | 595 | 1,483 | 587 | 1,438 | 552 | 1,376 | 514 | 8,516 | 573 |
| 70-79 | 1,187 | 744 | 1,121 | 707 | 1,105 | 697 | 1,076 | 674 | 1,054 | 656 | 864 | 534 | 6,407 | 668 |
| 80+ | 861 | 572 | 836 | 557 | 776 | 517 | 766 | 509 | 712 | 476 | 627 | 420 | 4,578 | 509 |
| Total | 6,752 | 288 | 6,927 | 293 | 6,717 | 281 | 6,912 | 286 | 6,817 | 279 | 6,369 | 258 | 40,494 | 280 |
| **Men+women** | |  |  |  |  |  |  |  |  |  |  |  |  |  |
| 0-4 | 0 | 0 | 0 | 0 | 0 | 0 | 0 | 0 | 0 | 0 | 0 | 0 | 0 | 0 |
| 5-9 | 0 | 0 | 2 | 1 | 0 | 0 | 3 | 1 | 0 | 0 | 3 | 1 | 8 | 0 |
| 10-14 | 15 | 5 | 10 | 3 | 4 | 1 | 9 | 3 | 9 | 3 | 10 | 3 | 57 | 3 |
| 15-19 | 59 | 19 | 69 | 22 | 48 | 15 | 57 | 18 | 68 | 21 | 69 | 21 | 370 | 19 |
| 20-29 | 597 | 106 | 613 | 107 | 610 | 103 | 672 | 111 | 646 | 104 | 665 | 103 | 3,803 | 106 |
| 30-39 | 1,214 | 177 | 1,215 | 179 | 1,204 | 178 | 1,164 | 173 | 1,225 | 182 | 1,229 | 183 | 7,251 | 179 |
| 40-49 | 1,774 | 268 | 1,971 | 293 | 2,081 | 303 | 2,090 | 299 | 2,122 | 299 | 2,048 | 283 | 12,086 | 291 |
| 50-59 | 2,952 | 489 | 3,133 | 518 | 3,071 | 505 | 3,193 | 520 | 3,119 | 503 | 3,064 | 488 | 18,532 | 504 |
| 60-69 | 3,174 | 713 | 3,469 | 741 | 3,569 | 731 | 3,596 | 711 | 3,595 | 689 | 3,383 | 629 | 20,786 | 701 |
| 70-79 | 2,330 | 802 | 2,346 | 808 | 2,265 | 778 | 2,284 | 776 | 2,214 | 743 | 1,924 | 639 | 13,363 | 757 |
| 80+ | 1,467 | 636 | 1,425 | 616 | 1,363 | 588 | 1,297 | 556 | 1,294 | 555 | 1,105 | 472 | 7,951 | 570 |
| Total | 13,582 | 291 | 14,253 | 303 | 14,215 | 298 | 14,365 | 297 | 14,292 | 292 | 13,500 | 273 | 84,207 | 292 |

| **Table S5** Incident users and incidence rate (per 100,000 person years) of insulin only use  2006-2010 in Norway (no A10 previous 24 months and no A10B 12 months after first insulin prescription dispensed) | | | | | | | | | | |
| --- | --- | --- | --- | --- | --- | --- | --- | --- | --- | --- |
|  | 2006 | | 2007 | | 2008 | | 2009 | | 2010 | |
| Age (years) | N | Incidence rate | N | Incidence rate | N | Incidence rate | N | Incidence rate | N | Incidence rate |
|  |  |  |  |  |  |  |  |  |  |  |
| **Men** |  |  |  |  |  |  |  |  |  |  |
| 0-4 | 27 | 20 | 17 | 13 | 31 | 23 | 31 | 22 | 27 | 19 |
| 5-9 | 59 | 38 | 50 | 33 | 51 | 34 | 59 | 39 | 49 | 32 |
| 10-14 | 67 | 42 | 82 | 51 | 85 | 53 | 87 | 54 | 92 | 57 |
| 15-19 | 54 | 34 | 52 | 32 | 57 | 35 | 67 | 41 | 66 | 40 |
| 20-29 | 96 | 34 | 92 | 32 | 101 | 34 | 97 | 31 | 89 | 28 |
| 30-39 | 101 | 29 | 98 | 28 | 104 | 30 | 85 | 25 | 94 | 27 |
| 40-49 | 85 | 25 | 90 | 26 | 99 | 28 | 101 | 28 | 95 | 26 |
| 50-59 | 83 | 27 | 94 | 31 | 104 | 34 | 91 | 29 | 83 | 26 |
| 60-69 | 95 | 43 | 102 | 44 | 107 | 44 | 106 | 42 | 103 | 39 |
| 70-79 | 57 | 43 | 68 | 52 | 70 | 53 | 74 | 55 | 71 | 52 |
| 80+ | 32 | 40 | 48 | 59 | 36 | 44 | 41 | 49 | 44 | 53 |
|  | 756 | 33 | 793 | 34 | 845 | 36 | 839 | 35 | 813 | 33 |
| **Women** | |  |  |  |  |  |  |  |  |  |
| 0-4 | 19 | 15 | 24 | 19 | 24 | 18 | 32 | 24 | 23 | 17 |
| 5-9 | 58 | 39 | 48 | 33 | 51 | 35 | 52 | 36 | 52 | 36 |
| 10-14 | 63 | 41 | 73 | 48 | 73 | 48 | 70 | 46 | 66 | 43 |
| 15-19 | 38 | 25 | 28 | 18 | 37 | 24 | 52 | 33 | 28 | 18 |
| 20-29 | 90 | 32 | 101 | 36 | 138 | 48 | 116 | 39 | 118 | 38 |
| 30-39 | 157 | 47 | 153 | 46 | 172 | 52 | 161 | 49 | 193 | 59 |
| 40-49 | 58 | 18 | 58 | 18 | 72 | 22 | 53 | 16 | 74 | 21 |
| 50-59 | 50 | 17 | 55 | 18 | 55 | 18 | 48 | 16 | 69 | 23 |
| 60-69 | 56 | 25 | 54 | 23 | 62 | 25 | 58 | 23 | 75 | 29 |
| 70-79 | 37 | 23 | 36 | 23 | 44 | 28 | 54 | 34 | 42 | 26 |
| 80+ | 44 | 29 | 49 | 33 | 55 | 37 | 49 | 33 | 44 | 29 |
|  | 670 | 29 | 679 | 29 | 783 | 33 | 745 | 31 | 784 | 32 |
| **Men+women** | |  |  |  |  |  |  |  |  |  |
| 0-4 | 46 | 18 | 41 | 16 | 55 | 21 | 63 | 23 | 50 | 18 |
| 5-9 | 117 | 39 | 98 | 33 | 102 | 34 | 111 | 37 | 101 | 34 |
| 10-14 | 130 | 41 | 155 | 49 | 158 | 50 | 157 | 50 | 158 | 50 |
| 15-19 | 92 | 30 | 80 | 25 | 94 | 30 | 119 | 37 | 94 | 29 |
| 20-29 | 186 | 33 | 193 | 34 | 239 | 41 | 213 | 35 | 207 | 33 |
| 30-39 | 258 | 38 | 251 | 37 | 276 | 41 | 246 | 37 | 287 | 43 |
| 40-49 | 143 | 22 | 148 | 22 | 171 | 25 | 154 | 22 | 169 | 24 |
| 50-59 | 133 | 22 | 149 | 25 | 159 | 26 | 139 | 23 | 152 | 25 |
| 60-69 | 151 | 34 | 156 | 33 | 169 | 35 | 164 | 32 | 178 | 34 |
| 70-79 | 94 | 32 | 104 | 36 | 114 | 39 | 128 | 43 | 113 | 38 |
| 80+ | 76 | 33 | 97 | 42 | 91 | 39 | 90 | 39 | 88 | 38 |
|  | 1,426 | 31 | 1,472 | 31 | 1,628 | 34 | 1,584 | 33 | 1,597 | 33 |

| **Table S6** Mean population used in calculation of incidence rates and prevalences of use of blood glucose-lowering drugs in Norway 2005-2011 | | | | | | | | |
| --- | --- | --- | --- | --- | --- | --- | --- | --- |
| Age | 2005 | 2006 | 2007 | 2008 | 2009 | 2010 | 2011 | Total |
|  |  |  |  |  |  |  |  |  |
| **Men** |  |  |  |  |  |  |  |  |
| 0-4 | 131,784 | 132,536 | 134,598 | 136,598 | 139,246 | 142,027 | 143,391 | 960,179 |
| 5-9 | 156,696 | 154,431 | 152,344 | 152,160 | 151,855 | 151,483 | 153,252 | 1,072,220 |
| 10-14 | 160,788 | 161,144 | 161,161 | 161,179 | 161,403 | 161,160 | 159,237 | 1,126,069 |
| 15-19 | 153,138 | 157,624 | 161,672 | 163,397 | 164,885 | 166,183 | 167,182 | 1,134,079 |
| 20-29 | 283,218 | 285,367 | 291,412 | 299,993 | 308,201 | 316,930 | 327,916 | 2,113,035 |
| 30-39 | 350,150 | 346,542 | 344,759 | 344,645 | 343,574 | 343,175 | 343,602 | 2,416,446 |
| 40-49 | 333,067 | 338,130 | 344,510 | 352,435 | 359,379 | 365,155 | 371,715 | 2,464,389 |
| 50-59 | 307,118 | 306,175 | 306,993 | 309,148 | 312,352 | 315,506 | 319,871 | 2,177,161 |
| 60-69 | 206,650 | 220,679 | 232,684 | 243,401 | 252,749 | 261,151 | 269,780 | 1,687,094 |
| 70-79 | 131,795 | 131,100 | 131,602 | 132,676 | 134,723 | 137,270 | 139,288 | 938,452 |
| 80+ | 78,701 | 80,280 | 81,091 | 81,732 | 82,842 | 83,660 | 84,629 | 572,932 |
| Total | 2,293,103 | 2,314,006 | 2,342,823 | 2,377,361 | 2,411,205 | 2,443,697 | 2,479,860 | 16,662,054 |
| **Women** |  |  |  |  |  |  |  |  |
| 0-4 | 126,347 | 127,051 | 128,688 | 130,258 | 132,359 | 134,677 | 135,815 | 915,194 |
| 5-9 | 148,814 | 147,153 | 145,744 | 145,720 | 145,601 | 145,393 | 146,876 | 1,025,300 |
| 10-14 | 151,924 | 152,223 | 152,585 | 152,610 | 152,900 | 153,000 | 151,613 | 1,066,854 |
| 15-19 | 145,373 | 149,923 | 152,956 | 154,722 | 155,749 | 155,919 | 156,601 | 1,071,242 |
| 20-29 | 276,717 | 277,958 | 282,561 | 289,600 | 297,436 | 306,631 | 315,843 | 2,046,746 |
| 30-39 | 340,384 | 337,478 | 334,060 | 331,583 | 329,704 | 328,848 | 327,834 | 2,329,889 |
| 40-49 | 320,775 | 323,945 | 328,290 | 333,702 | 339,898 | 345,330 | 351,160 | 2,343,098 |
| 50-59 | 297,332 | 296,943 | 297,340 | 299,326 | 302,000 | 304,737 | 308,564 | 2,106,241 |
| 60-69 | 211,727 | 224,459 | 235,474 | 244,621 | 252,790 | 260,446 | 267,913 | 1,697,428 |
| 70-79 | 161,720 | 159,442 | 158,618 | 158,429 | 159,536 | 160,781 | 161,691 | 1,120,214 |
| 80+ | 149,324 | 150,463 | 150,147 | 150,147 | 150,624 | 149,490 | 149,319 | 1,049,514 |
| Total | 2,330,434 | 2,347,036 | 2,366,461 | 2,390,716 | 2,418,595 | 2,445,249 | 2,473,228 | 16,771,717 |
| **Men+women** | |  |  |  |  |  |  |  |
| 0-4 | 258,130 | 259,586 | 263,286 | 266,856 | 271,605 | 276,703 | 279,206 | 1,875,372 |
| 5-9 | 305,510 | 301,584 | 298,088 | 297,879 | 297,456 | 296,875 | 300,128 | 2,097,519 |
| 10-14 | 312,712 | 313,366 | 313,746 | 313,788 | 314,302 | 314,160 | 310,850 | 2,192,923 |
| 15-19 | 298,510 | 307,547 | 314,627 | 318,119 | 320,634 | 322,102 | 323,783 | 2,205,321 |
| 20-29 | 559,935 | 563,325 | 573,973 | 589,593 | 605,636 | 623,561 | 643,759 | 4,159,781 |
| 30-39 | 690,534 | 684,020 | 678,818 | 676,228 | 673,278 | 672,023 | 671,436 | 4,746,335 |
| 40-49 | 653,842 | 662,075 | 672,800 | 686,136 | 699,276 | 710,484 | 722,875 | 4,807,487 |
| 50-59 | 604,450 | 603,118 | 604,333 | 608,474 | 614,352 | 620,243 | 628,434 | 4,283,402 |
| 60-69 | 418,377 | 445,138 | 468,158 | 488,022 | 505,539 | 521,597 | 537,693 | 3,384,522 |
| 70-79 | 293,515 | 290,541 | 290,219 | 291,105 | 294,259 | 298,051 | 300,978 | 2,058,666 |
| 80+ | 228,025 | 230,743 | 231,238 | 231,879 | 233,465 | 233,150 | 233,948 | 1,622,445 |
| Total | 4,623,537 | 4,661,041 | 4,709,284 | 4,768,077 | 4,829,800 | 4,888,946 | 4,953,088 | 33,433,771 |
