# Supplementary material for: No increase in new users of blood glucose-lowering drugs in Norway 2006–2011: a nationwide prescription database study
Source: BMC Public Health. 2014 May 29;14:520. doi: 10.1186/1471-2458-14-520 (PMC4045953; doi:10.1186/1471-2458-14-520)
Supplement: Additional file 2: Figure S1 — New users of insulins only among women aged 30-39 years in 2008. [file 1471-2458-14-520-S2.docx]

**Additional file 2: Figure S1** New users of insulins only among women aged 30-39 years in 2008
